# Radioprotective Effect of Alk(en)yl Thiosulfates Derived from Allium Vegetables against DNA Damage Caused by X-Ray Irradiation in Cultured Cells: Antiradiation Potential of Onions and Garlic

**DOI:** 10.1100/2012/846750

**Published:** 2012-07-31

**Authors:** Hye-Sook Chang, Daiji Endoh, Yushi Ishida, Hiroyuki Takahashi, Shuji Ozawa, Masanobu Hayashi, Akira Yabuki, Osamu Yamato

**Affiliations:** ^1^Laboratory of Clinical Pathology, Department of Veterinary Medicine, Kagoshima University, 1-21-24 Kohrimoto, Kagoshima 890-0065, Japan; ^2^Department of Veterinary Radiology, School of Veterinary Medicine, Rakuno Gakuen University, 582 Midorimachi, Bunkyodai, Hokkaido 069-8501, Ebetsu, Japan; ^3^Information Systems Department, Industrial Research Institute, Hokkaido Research Organization, Kita-19 Nishi-11, Hokkaido 060-0819, Sapporo, Japan; ^4^Laboratory of Natural Products Chemistry, Department of Sustainable Agriculture, Rakuno Gakuen University, 582 Midorimachi, Bunkyodai, Hokkaido 069-8501, Ebetsu, Japan

## Abstract

To evaluate a radioprotective effect of sodium *n*-propyl thiosulfate (NPTS) and sodium 2-propenyl thiosulfate (2PTS) derived from onions and garlic, respectively, rat hepatoma H4IIE cells and mouse lymphoma L5178Y cells were preincubated with each of these compounds for 48 hours at 37°C before receiving 10 Gy of X-ray irradiation. Cell damage caused by the irradiation was quantified as comet tail moment, which represents the degree of DNA damage. X-ray-induced DNA damage was significantly decreased in both H4IIE and L5178Y cells by micromolar concentrations of NPTS and 2PTS compared with the control without the compounds. The protective effect was more potent with 2PTS than NPTS. Onions and garlic have antiradiation potential.

## 1. Introduction

Radiation exposure, either accidentally or during radiotherapy and radio imaging, has deterministic or stochastic effects on living systems. The development of effective and nontoxic radioprotective agents is therefore of considerable interest, and a large number of chemical and biological agents have been screened over the past decade [1, 2]. As yet, however, there is not a single agent available which meets all the prerequisites of an ideal radio protector, that is, produces no cumulative or irreversible toxicity, offers effective long-term protection, possesses a shelf life of 2–5 years, and can be easily administered [1].

Garlic extract and its chemical constituents such as allicin, alliin, and diallyl sulfide have inhibitory effects on chemical mutagenesis and carcinogenesis [3–5]. Furthermore, experiments* in vitro* using *Salmonella* tester strains and Chinese hamster ovary cells showed that garlic exerts an antimutagenic effect against gammaradiation possibly through the scavenging of free radicals [4]. Other researchers also demonstrated a radioprotective effect of garlic against tissue damage in liver and lung, reducing the radiation-induced mortality in albino rats [6]. Based on these observations, some constituents in Allium vegetables such as onions and garlic are expected to be radioprotective.

**Figure 1:**
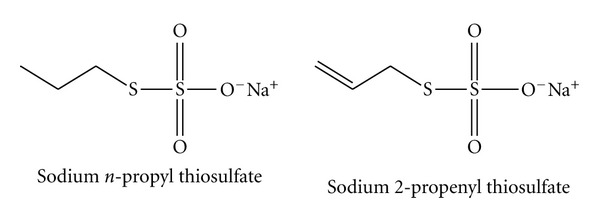
Structure of sodium *n*-propyl thiosulfate (NPTS) and sodium 2-propenyl thiosulfate (2PTS).

Two alk(en)yl thiosulfates (Figure 1), sodium *n*-propyl thiosulfate (NPTS) and sodium 2-propenyl thiosulfate (2PTS), isolated from onions (*Allium cepa*) and garlic (*A. sativum*), respectively, were originally identified as causative agents of onion- and garlic-induced hemolytic anemia in dogs [7–10]. To date, however, NPTS and 2PTS have been shown to have a number of beneficial properties for humans, that is, an inhibitory effect on tumor cell growth [11–13], an immune-enhancing effect on polymorphonuclear leukocytes [14], an antiaggregatory effect on platelets [14, 15], and an inductive effect on phase II detoxification enzymes [16]. In contrast, there has not been any report of anemia associated with the consumption of onions or garlic in humans.

In view of the usefulness of these Allium vegetables and the multifunction of these alk(en)nyl thiosulfates for human health, the present study was undertaken to evaluate a possible radioprotective effect of NPTS and 2PTS against X-ray-induced DNA damage in cultured mammalian cells. In addition, the radioprotective effect of these compounds was compared with that of lignan, recently reported to have a radioprotective effect in cultured cells [17] and in mice [18].

## 2. Materials and Methods

All experiments in the present study were carried out in laboratories specific for radiation experiments in the Rakuno Gakuen University under the supervision of a radiology specialist (D. Endoh, one of the authors).

### 2.1. Chemicals and Reagents

 NPTS and 2PTS were synthesized and purified according to a method described previously [7, 10]. Lignan-containing flaxseed extract was prepared as reported previously [19]. Normal melting-point agarose (NMA) was obtained from the Nacalai Tesque (Kyoto, Japan) and low melting-point agarose (LMA) from Funakoshi (Tokyo, Japan).

### 2.2. Cell Culture and Treatments

The rat hepatoma H4IIE cell line was purchased from the Dainippon Sumitomo Pharma (Osaka, Japan). H4IIE cells were grown in Dulbecco's modified eagle's medium supplemented with 10% fetal bovine serum, 100 U/mL penicillin, and 100 *μ*g/mL streptomycin. The mouse lymphoma L5178Y cell line was provided by the Riken Bio Resource Center (Tsukuba, Japan). L5178Y cells were grown in RPMI 1640 medium supplemented with 10% heat-inactivated horse serum, 100 U/mL penicillin, and 100 *μ*g/mL streptomycin. NPTS and 2PTS were dissolved in culture medium and adjusted to the concentrations required for the experiments. Lignan-containing flaxseed extract was diluted with culture medium and adjusted to the required concentration. The cells were seeded at an initial density of 7 × 10^5^ cells into a 25 cm^2^ flask, preincubated for 24 hours at 37°C, and then exposed to final concentrations (1–50 *μ*M) of NPTS, 2PTS, or 2.5 mg/mL of lignan-containing flaxseed extract for 48 hours at 37°C. Control cells were exposed to nothing.

### 2.3. Comet Assay

The comet assay was carried out according to procedures reported previously [20, 21], with some modifications. A microscope slide was precoated in 1% NMA and allowed to dry at room temperature. The cell suspension was mixed with an equal volume of 2% LMA at 37°C. Sixty microliter of the LMA cell suspension was rapidly pipetted over an NMA precoated slide and covered with a coverslip as the cells were spread homogeneously. After solidification of the LMA cell suspension for 15 min at 4°C, the coverslip was gently removed. On the solidified LMA cell suspension, 100 *μ*L of cell-free LMA was layered, covered with a coverslip, and then allowed to solidify for 20 min at 4°C. After removal of the coverslip, the slide for irradiation experiments was exposed to a 10 Gy dose of X-rays. Following completion of the irradiation, the slide was immediately immersed into alkaline lysis solution containing 1 M NaCl, 0.03 M NaOH, and 0.1% sodium sarcosinate for 30 min at 4°C, being protected from light. Next the slide was placed in electrophoresis solution (pH 13) containing 0.3 M NaOH and 2 mM EDTA, and electrophoresis was conducted for 25 min at 25 V and 400 mA. After the electrophoresis, the slide was neutralized in neutralization buffer (pH 7.4) containing 154 mM NaCl, 1 mM CaCl_2_, 0.5 mM MgCl_2_, and 100 mM Tris, for 20 min, and stained with PicoGreen (Invitrogen, Eugene, OR, USA) diluted to 200-fold in the neutralization buffer, and photographed using an inspection device (Bioplorer FJ-VKH-01; Koyo Sangyo, Tokyo, Japan; Panasonic Ecology Systems, Kasugai-shi, Japan). Image analysis of the photograph was performed using originally developed software (Hokkaido Industrial Research Institute, Sapporo, Japan) on cells selected randomly by the software (Japanese Patent no. 4355832). The extent of DNA damage was quantified as the tail moment of 50–100 individual cells per treatment.

### 2.4. X-Ray Irradiation

The cells were irradiated to a dose of 10 Gy using an X-ray generator (Model MBR-1520R, Hitachi, Tokyo, Japan) operated at 150 kVp and 20 mA with 0.5 mm Cu and 0.5 mm Al filtration. The source-to-surface distance was 30 cm, and the dose rate was approximately 1.12 Gy/min.

### 2.5. Statistical Analyses

All data are presented as the mean ± standard deviation of the tail moment in 50–100 cells selected randomly by the software for image analysis. For comparisons between groups, data were analyzed by a one-way factorial analysis of variance with post hoc tests (Tukey's method). Differences were considered significant at *P* < 0.05.

## 3. Results

**Figure 2:**
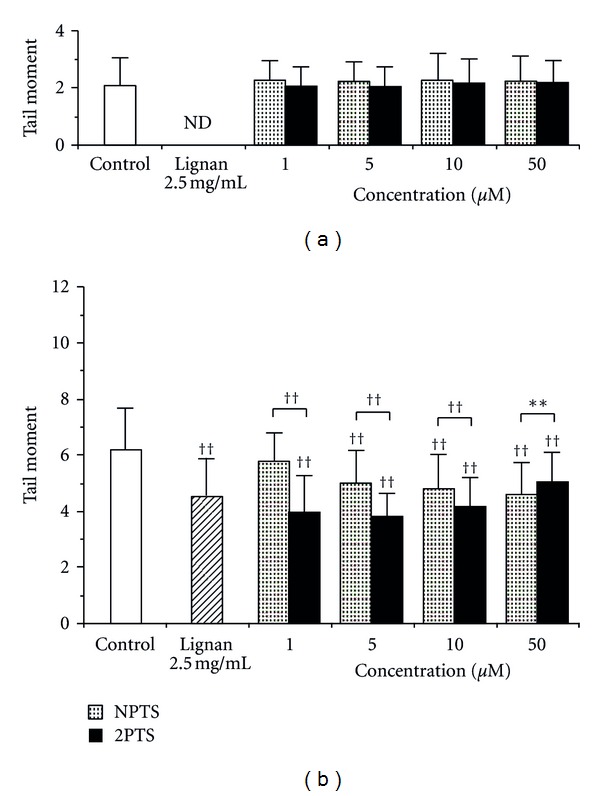
The effect of alk(en)yl thiosulfates on X-ray-induced DNA damage in rat hepatoma H4IIE cells. Cells were pretreated with different concentrations (1–50 *μ*M) of sodium *n*-propyl thiosulfate (NPTS), sodium 2-propenyl thiosulfate (2PTS), or 2.5 mg/mL lignan-containing flaxseed extract for 48 hours at 37°C before X-ray irradiation (10 Gy). Control cells (white column) were not exposed to any compound. The experiments were divided into nonirradiated (a) and X-ray-irradiated (b) groups. The assessment of DNA damage was carried out on 50–100 individual cells per treatment using a comet assay. Data were analyzed by a one-way factorial analysis of variance with post hoc tests (Tukey's method). **P < 0.01 and ^††^ *P* < 0.001 versus control or between groups. ND: not determined.

The results of the comet assay using H4IIE cells are shown in Figure 2. NPTS and 2PTS did not change comet tail moment in H4IIE cells without irradiation (Figure 2(a)). In control cells without any compound, tail moment was increased 3.0-fold by irradiation (Figure 2(b)) compared with that in control cells without irradiation shown in Figure 2(a). Tail moment was significantly reduced by 26% by treatment with lignan, compared with control cells with irradiation (Figure 2(b)). In cells treated with NPTS, tail moment was decreased dose-dependently and by a maximum of 25% at 50 *μ*M. The values at 5–50 *μ*M NPTS were significantly lower than those in the control. In 2PTS, the values at all concentrations were significantly lower than those in the control, but the change was not dose dependent: the lowest (by 39%) was at 5 *μ*M and the highest (by 19%) at 50 *μ*M. In comparisons between NPTS and 2PTS, tail moment was significantly lower in 2PTS than that in the latter at 1–10 *μ*M, but significantly higher at 50 *μ*M.

**Figure 3:**
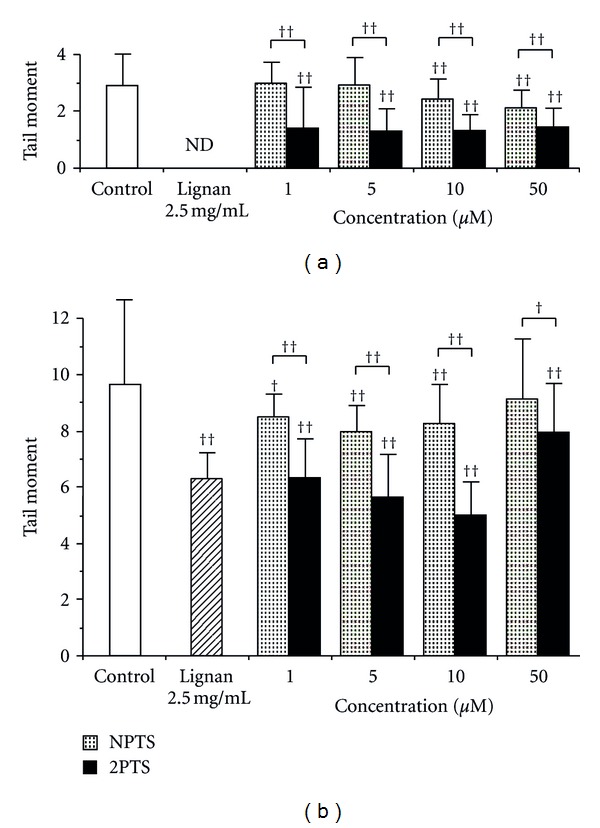
The effect of alk(en)yl thiosulfates on X-ray-induced DNA damage in mouse lymphoma L5178Y cells. Cells were pretreated with different concentrations (1–50 *μ*M) of sodium *n*-propyl thiosulfate (NPTS), sodium 2-propenyl thiosulfate (2PTS), or 2.5 mg/mL lignan-containing flaxseed extract for 48 hours at 37°C before the X-ray irradiation (10 Gy). Control cells (white column) were not exposed to any compound. The experiments were divided into nonirradiated (a) and X-ray-irradiated (b) groups. The assessment of DNA damage was carried out on 50–100 individual cells per treatment using a comet assay. Data were analyzed by a one-way factorial analysis of variance with post hoc tests (Tukey's method). ^†^ *P* < 0.005 and ^††^ *P* < 0.001 versus control or between groups. ND: not determined.

The results in the comet assay using L5178Y cells are shown in Figure 3. Even in nonirradiation experiments, tail moment was significantly reduced at 10 and 50 *μ*M NPTS and at all concentrations of 2PTS (Figure 3(a)). The value was significantly lower in 2PTS than in NPTS at all concentrations. In irradiation experiments (Figure 3(b)), the results were similar to those in H4IIE cells shown in Figure 2(b). In control cells, tail moment was increased 3.3-fold by irradiation (Figure 3(b)). Tail moment was significantly reduced by 35% by treatment with lignan. Both NPTS and 2PTS reduced tail moment, but the changes were not dose dependent. In NPTS, tail moment was significantly decreased at 1–10 *μ*M and the lowest value (by 17%) was at 5 *μ*M. In 2PTS, tail moment was significantly decreased at all concentrations and the lowest value (by 48%) was at 10 *μ*M. Tail moment was significantly lower in 2PTS than that in NPTS at all concentrations.

## 4. Discussion

In the present study, we investigated a radioprotective effect of two alk(en)yl thiosulfates and lignan against DNA damage caused by X-ray irradiation in 2 kinds of cultured mammalian cells. Based on the results, it was demonstrated that a 48-hour culture with NPTS and 2PTS reduced the degree of DNA damage caused by subsequent irradiation in both cell lines, like lignan, an effect of which had been reported previously [17, 18]. The radioprotective effect of NPTS and 2PTS is consistent with findings from studies *in vitro*, which demonstrated an antimutagenic effect of garlic against gammaradiation in *Salmonella* and Chinese hamster ovary cells [4].

Since free radicals play an important role in irradiation-induced damage, the underlying radioprotective mechanism of a majority of radioprotective compounds has been linked, either directly or indirectly, to their antioxidative capabilities by scavenging free radicals responsible for DNA damage [22]. Garlic oil is rich in sulfhydryl compounds that are capable of protecting against tissue-damaging effects of irradiation [6]. The mechanism of the protective effect by sulfhydryl compounds is considered to be free radical extinction. Diallyl trisulfide, a principal constituent of garlic oil, significantly induced the production of glutathione peroxidase, one of the most important antioxidative enzymes in living systems [23]. Other researchers investigated effects of onions on red cell antioxidants such as superoxide dismutase, catalase, glucose-6-phosphate, and reduced glutathione (GSH) by feeding onion soup to dogs [24]. In this experiment, there was a transient decrease in the antioxidants, but values then began to increase progressively toward a peak, before returning to normal. In our previous experiment *in vivo* using NPTS, the concentration of erythrocyte GSH was significantly increased after the oral administration of NPTS to dogs although NPTS acts as an oxidant-inducing hemolytic anemia in dogs [8]. These observations suggest that the body's antioxidation system could be stimulated by pre-exposure to certain worthwhile oxidants, which enhances endogenous protective mechanisms against oxidative stress. There is considerable evidence that Allium vegetables prevent cancer in humans [25–28]. The radioprotective effect of NPTS and 2PTS may be one of the mechanisms of cancer prevention.

In the present study, significantly effective radioprotection was not always obtained dose dependently. The radioprotective effect at relatively high concentrations of alk(en)yl thiosulfates seemed to become weaker rather than stronger. In H4IIE cells, 2PTS showed higher levels of DNA damage at 10 and 50 *μ*M than at 1 and 5 *μ*M (Figure 2(b)). Likewise, in L5178Y cells, both NPTS and 2PTS showed higher levels of DNA damage at 50 *μ*M than those at the other lower concentrations (Figure 3(b)). Therefore, it is thought that overexposure to alk(en)yl thiosulfates could result in excessive oxidative stress rather than the level of stress appropriate for stimulating the body's antioxidation system. In other words, NPTS and 2PTS exert an antioxidative effect by stimulating the antioxidation system at low concentrations, but as their concentrations increase, their natural characteristics as oxidants become evident and the radioprotective effect might be offset. In fact, NPTS and 2PTS have an antitumor effect through the induction of apoptosis initiated by oxidative stress at relatively high concentrations (>50 *μ*M) [11].

Computed tomography (CT) is one of the largest contributors to man-made irradiation doses in medical populations, and its use continues to grow rapidly all over the world. The principal concern regarding irradiation exposure is that the subject may develop malignancies. Many studies have shown that organ doses associated with routine diagnostic CT scans are similar to the low-dose range of irradiation received by atomic-bomb survivors, and the US Food and Drug Administration estimates that a CT examination with an effective dose of 10 mSv may be associated with an increased chance of developing fatal cancer for approximately one in 2000 patients [29]. In addition, there is a risk of radioactive pollutants being released into the environment by serious accidents at nuclear power plants, as happened at Fukushima in 2011 and Chernobyl in 1986.

We believe that the daily intake of Allium vegetables such as onions and garlic containing alk(en)yl thiosulfates and other effective compounds could be beneficial to decrease toxic effects of irradiation.
